# Uncovering the Sex-Specific Endocrine Responses to Reproduction and Parental Care

**DOI:** 10.3389/fendo.2021.631384

**Published:** 2021-11-11

**Authors:** Suzanne H. Austin, Jesse S. Krause, Rechelle Viernes, Victoria S. Farrar, April M. Booth, Rayna M. Harris, Frédéric Angelier, Candice Lee, Annie Bond, John C. Wingfield, Matthew M. MacManes, Rebecca M. Calisi

**Affiliations:** ^1^ Department of Neurobiology, Physiology, and Behavior, University of California Davis, Davis, CA, United States; ^2^ Department of Biology, University of Nevada, Reno, NV, United States; ^3^ Centre d’Etudes Biologiques de Chizé, CNRS, La Rochelle Université, UMR 7372, Villiers en Bois, France; ^4^ Department of Molecular, Cellular and Biomedical Sciences, The University of New Hampshire, Durham, NH, United States

**Keywords:** reproduction, birds, prolactin, corticosterone, sex steroids

## Abstract

Hormones mediate physiological and behavioral changes in adults as they transition into reproduction. In this study, we characterize the circulating levels of five key hormones involved in reproduction in rock doves (*Columba livia*): corticosterone, progesterone, estradiol, testosterone, and prolactin using univariate and multivariate approaches. We show similar patterns as previous studies in the overall patterns in circulating levels of these hormones, i.e., testosterone (males) and estradiol (females) high during nest-building or egg-laying, prolactin increasing at mid-incubation and peaking at hatching (both sexes), and elevated corticosterone levels in later incubation and early nestling development. In our investigation of hormone co-variation, we find a strong correlation between prolactin and corticosterone across sampling stages and similarities in earlier (early to mid-incubation) compared to later (late incubation to nestling d9) sampling stages in males and females. Finally, we utilized experimental manipulations to simulate nest loss or altered caregiving lengths to test whether external cues, internal timing, or a combination of these factors contributed most to hormone variation. Following nest loss, we found that both males and females responded to the external cue. Males generally responded quickly following nest loss by increasing circulating testosterone, but this response was muted when nest loss occurred early in reproduction. Similar treatment type, e.g., removal of eggs, clustered similarly in hormone space. These results suggest internal drivers limited male response early in reproduction to nest loss. In contrast, circulating levels of these hormones in females either did not change or decreased following nest manipulation suggesting responsiveness to external drivers, but unlike males, this result suggests that reproductive processes were decreasing.

## Introduction

The parental care period is a critical life-history sub-stage that directly influences individual fitness (1). Avian parenting typically involves adults building nests where they lay their eggs, engage in contact-incubation of eggs, and brood and feed their young (depending on the developmental mode of the offspring) until independence. These complex behaviors require that adults make trade-offs between self-maintenance and offspring care (2, 3). Hormones mediate many of these physiological and behavioral changes during reproduction (4). Variation in multiple hormones, and their role in reproduction, has been described through a series of descriptive and experimental studies [reviewed in (5)]. Here, we briefly summarize the hormonal regulation of reproductive behavior.

### Initiation of Reproduction

The endocrine system is critically involved in the initiation and regulation of reproductive and parental behaviors (6). gonadotropin-releasing hormone I, GnRH, produced in the hypothalamus stimulates the production and release of luteinizing hormone (LH) and follicle-stimulating hormone (FSH) in the anterior pituitary (7). LH and FSH then circulate through the bloodstream to the gonads where they induce gonadal growth and steroidogenesis of testosterone, estradiol, and progesterone (6, 7).

### Courtship and Nest-Building

Male courtship behavior is linked to androgens, such as circulating testosterone. Following exposure to a potential mate, circulating testosterone increased within 4-hours (8) and was maintained at high levels during courtship and also promoted spermatogenesis. Testosterone levels drop at the onset of incubation (6, 8). Male nest-building behavior is predominately dependent on female cues (9). However, administration of exogenous progesterone can stimulate nest-building behavior even though its circulating levels remain generally low in males during courtship and nest-building (10, 11).

Studies that utilized exogenous hormones found that nest-building behavior in females is regulated by estradiol, vasoactive intestinal peptide (VIP), and FSH (6, 9). Estradiol is particularly important for the initiation of female courtship, nest-building, and the onset of incubation as evidenced in studies using ovariectomized birds (12, 13).

### Laying

Prior to egg-laying, blood estrogen levels peak and may act to stimulate the production of blood calcium-binding proteins (6). In birds, progesterone and estrogen induce an LH surge and ovulation (14, 15). The LH surge is also associated with muscle contractions in the oviduct required for oviposition (6). The joint actions of mesotocin and arginine vasotocin increase in the blood which induces oviposition and uterine contractions in birds (16). Prolactin, typically more associated with incubation, experiences a ‘transient increase’ in intact females during ovulation that corresponds with the onset of incubation and at clutch completion when it is thought to terminate laying (7). In most avian species, prolactin is critical to the onset and maintenance of incubation behavior [reviewed in (17, 18)]. Brood patch formation, used to contact-incubate eggs, is induced by estrogen (defeathering and vascularization) and prolactin (edema) (19). The degree to which birds develop brood patches varies by species.

### Incubation

Following clutch completion, gonadotropins (LH, FSH) and gonadal steroids (testosterone, estradiol, progesterone) decrease and remain low throughout the incubation and chick-rearing stages in both sexes (6, 20). Ovaries stop recruiting follicles for ovulation and decrease in size while maintaining a mature resting state after the clutch is laid. A number of these follicles are partially developed and can be rapidly recruited into final maturation leading to ovulation when required. The hormone prolactin, which has an antagonistic relationship with corticosterone, appears to regulate the onset and maintenance of incubation in most bird species (17, 18). Prolactin alone does not instigate incubation behaviors but must be coupled with estradiol and progesterone (6). Brood patch formation occurs prior to incubation in most species and has been attributed to increased estradiol, that causes defeathering and vascularization, and VIP and prolactin, that are associated with the development of the brood patch edema (21). In males, progesterone, along with other sex steroids, has also been attributed to the onset of incubation behavior (13). While prolactin is associated with the onset and maintenance of incubation in most birds, in pigeons and doves, prolactin levels do not begin to increase until later incubation. The delayed increase in prolactin in doves and pigeons is related to the production of crop milk in both sexes, which prolactin mediates (22). Columbids begin producing crop milk several days prior to hatching in preparation for offspring feeding. Rock pigeons have low vascularization of their defeathered brood patch, but do not form an edema (23). Lack of edema could indicate patch development is estrogen and not prolactin dependent in this species.

### Chick-Rearing and Brooding

In many precocial species, prolactin begins to decline soon after hatching whereas, in species with altricial young, prolactin remains elevated during the nestling period and then declines with chick age [reviewed in (17, 18)]. In pigeons and doves, prolactin levels correspond to the period of crop milk production (6). Prolactin has been associated with parental effort, brooding, and feeding rate in birds (13, 24, 25). Corticosterone (CORT) is a metabolic hormone that, like prolactin, increases during parental care because of the higher metabolic demands of parental care (13, 20, 24). In doves and pigeons, prolactin may also contribute to a hyperphagic parental response associated with crop milk production for chicks (7, 26). Gonadotropins and sex steroids generally remain low throughout parental care (6), which is thought to be related to high circulating prolactin inhibiting GnRH.

### Hormonal and Behavioral Response to External Stimuli

There is an extensive literature on the influence of external stimuli on breeding behavior and hormone synthesis [reviews can be found in (8, 27)]. As previously discussed, the presence of a potential mate can stimulate courtship behavior and sex steroid production in non-photorefractory males (8). Males exposed to a preovulatory female increased testosterone while those exposed to an incubating female decreased testosterone (28). While testosterone normally remains low during parental care [reviewed in (13)], this appears to be dependent on female behavior (29). If females maintain typical courtship behaviors during incubation, male androgens remain high, which suggests that female behavior, not breeding stage, drives male androgen levels (29). Males, too, can stimulate female receptiveness to breed. Exposure to a potential mate and nesting materials increased circulating LH in females (13).

Nest contents and their loss also influences parental behavior and hormone synthesis. When nests and eggs were removed during mid- to late-incubation, parents stopped incubating eggs and circulating prolactin levels decreased (30, 31). Exposure to nestlings can increase circulating prolactin levels (32, 33). Prolactin levels can also increase in response to experimentally lengthened incubation or brooding periods (25, 34). Thus, exposure to reproductively competent mates and alterations to the condition of the nest environment can influence circulating hormone levels.

**Figure 1:**
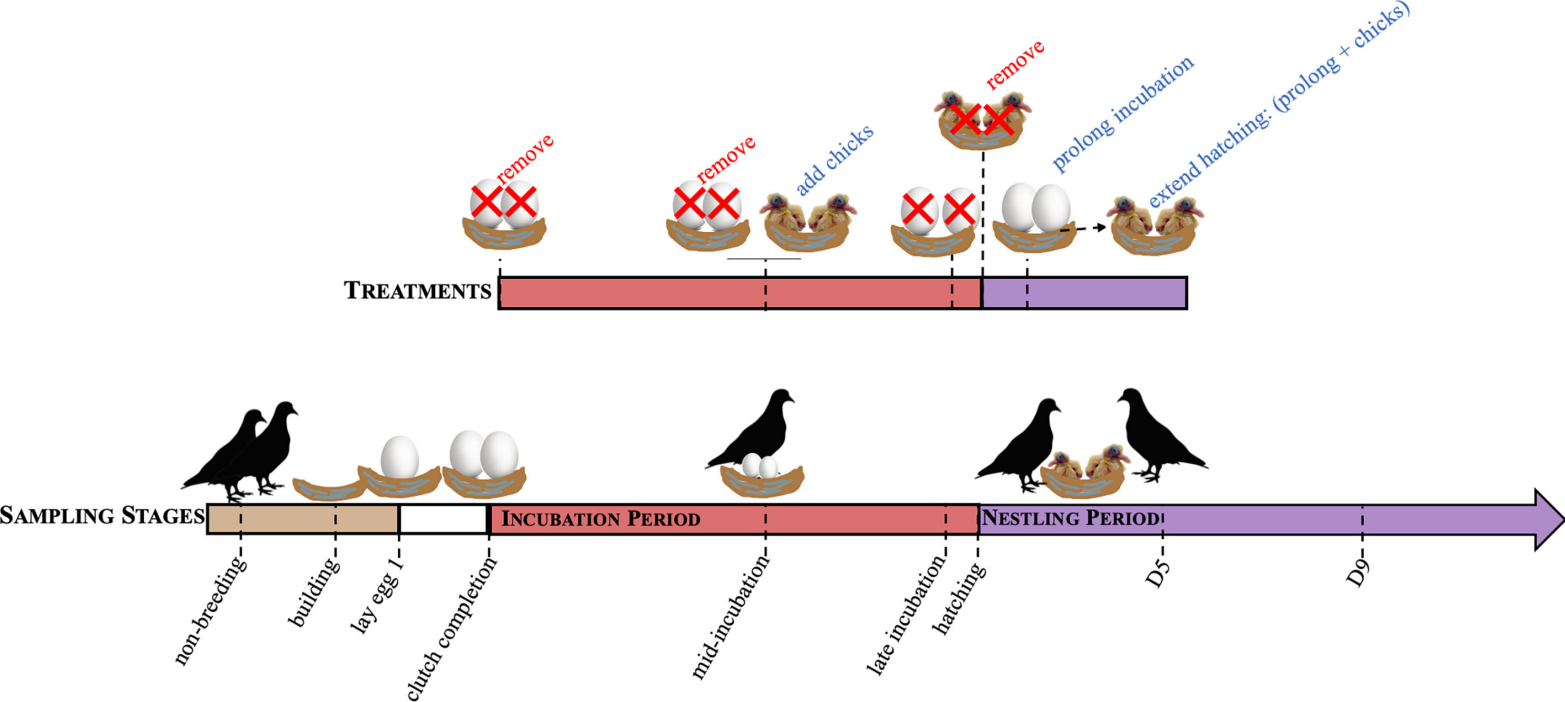
A schematic of the sampling time points to characterize hormones over reproduction (sampling stages) and their paired treatments. Sampling time points included, non-breeding, nest building, laying, clutch completion, mid-incubation (incubation d9), late incubation (incubation d17), hatch, nestling d5 (D5), and nestling d9 (D9). Concordant treatment groups include removing eggs at clutch completion, mid-incubation, and late incubation, and chicks at hatching to simulate nest loss. Adding chicks at mid-incubation decreased the length of the incubation period and incubation period was prolonged by 3 days (to d21) or hatching extended by adding chicks following prolongation.

**Figure 2:**
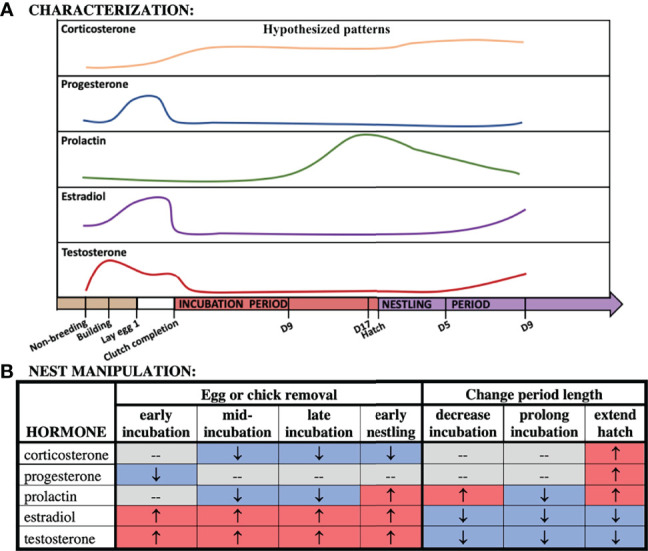
Hypothesized patterns of hormones across our time points **(A)** and following nest manipulation **(B)**.

While substantial information is known about circulating hormones and how they function during reproduction, few studies have addressed how hormones co-vary in males and females over multiple reproductive sub-stages or how these hormones are regulated. In this study, we characterized the profiles of five hormones (corticosterone, progesterone, estradiol, testosterone, and prolactin) across the reproductive cycle in rock doves (*Columba livia*). We sampled breeding pairs during non-breeding, nest-building, egg-laying, clutch completion, mid-incubation (d9), late incubation (d17), and chick-rearing at hatch and nestling d5 and d9 (**Figure 1**) to better understand how hormonal profiles vary throughout reproduction in males and females. Hypothesized patterns across breeding time points can be found in **Figure 2**. We also used a multivariate analysis in order to understand how variation in hormones cluster by stage and sex. For logistical reasons, investigations of co-variation in multiple hormones typically compare hormone levels across individuals, or if in the same individuals, only compare a couple of hormones. Because we were able to run assays for almost all hormones from the same individual samples, we were able to characterize co-variation of four different hormones in males and females. Covariation of these hormones across the breeding cycle allows us to better understand hormone inter-relationships in circulating levels across sampling stages.

In addition, we manipulated the presence of offspring to test whether the endocrine dynamics observed are driven internally (i.e., a biological clock schedule) or externally (the presence or absence of eggs or chicks) (**Figure 1**). We did this by conducting a series of experimental manipulations to test how environmental cues influence circulating hormone levels by removing eggs and chicks (at clutch completion, mid-incubation, late incubation, and hatch), decreasing (to mid-incubation, d9) and increasing the length of the incubation period (incubation increased to 21 days), and extending hatching (21d incubation + 1d of chick-rearing). If the level of a circulating hormone is externally driven, then we hypothesized that its concentration would change following a manipulation like egg or nestling removal. However, if hormone levels were controlled by internal timing, then we hypothesized that the hormone concentrations would not change in response to the external manipulation. While the absence of a hormone response may indicate internal timing, it may also mean that the manipulation did not adequately target a particular hormone. For instance, we would not expect that progesterone would change in response to removing the nest contents because progesterone is generally low during the breeding cycle except during egg-laying. Thus, we could only interpret the role of internal drivers based on how the trajectory of the hormone changed following external manipulation or its known biological actions. This means that if hormone levels responded to external manipulations, but the response was muted or enhanced at certain stages following a similar treatment, that internal timing may be driving these differences. We again used correlational and multivariate analyses to assess how hormones co-vary in response to experimental manipulation.

## Methods

### Study Species

Rock doves are a socially monogamous species where both sexes participate in all stages of parental care (35, 36). Following courtship, females lay a clutch of 1-2 eggs (35). In our captive population, laying occurs over 3 days with a 1-day gap between the first and second eggs. After the first egg is laid, eggs are nearly constantly incubated by both adults for 18.5 days. Adults share incubation and coordinate their attendance periods using time-specific ‘shifts’: females incubate from approximately mid-afternoon to mid-morning whereas males incubate from mid-morning to mid-afternoon (35, SHA personal observation). Both parents initially feed nestlings an energy rich secretion produced from the epithelial lining of their crops, known as crop milk. Males tend to feed nestlings more frequently than females while females typically brood the nestlings more than males, at least until they can thermoregulate at ~ 9-days post-hatching (35). As chicks age, parents begin integrating a regurgitant of seeds with crop milk. Crop milk production tends to cease at ~ 9-days after hatching (35) though there is variation in cessation of production (37). While both parents contribute to chick-rearing, males generally invest more in feeding chicks than females, particularly if a new breeding attempt is initiated (35). Nestlings fledge at approximately 28-days of age but will stay with the parents after independence, sometimes for up to a month post-fledging (35). In optimal conditions, rock doves can overlap clutches while nestlings are still in the nest; thus, parental behavior and physiology can overlap the sub-stages of clutch initiation, incubation, and chick-rearing. To facilitate this, parents split their efforts: males invest more in the later portion of one brood while females invest more in starting a new clutch.

### Housing

We housed domestic rock doves at the University of California, Davis, in large semi-enclosed outdoor aviaries (1.5 x1.2 x2.1 meters). Each aviary had approximately 8 sexually mature adult pairs plus some dependent offspring. We provided food and water *ad libitum*. Birds were exposed to natural light, which was augmented with artificial lights set to a 14L:10D cycle. All birds collected in this study were sexually mature and <2 years old.

### Nest Monitoring Methods

Nests were monitored daily to determine the reproductive stage of the pairs (see **Figure 1**) while determination of early stages of breeding (i.e., nest-building and egg laying) required behavioral observers by trained observers. Here, we noted behaviors indicative of a pair bonding in doves (e.g., nest-building, nuptial feeding, allopreening, copulation; see 35). During regular nest checks, we noted the identity of the parent(s) attending the nest by their unique color band combination, the stage of the nest (building, laying, incubating, nestling) the number and disposition of the eggs (intact, pipping, hatching) and the number of the chicks. From this information, we were able to determine the age of eggs (*where* clutch initiation = d1), the length of the incubation period, and when nestlings hatched and their age (*where* first hatch = d1).

### Animal Collections Methods

To control for circadian oscillations, birds were collected between 0900-1200 (PST) following animal care and handling protocols (UC Davis IACUC permit #18895). We sampled a total of 160 pairs (16 groups with 10 males and females/group). Once a pair was deemed to be at the appropriate breeding time point, they were captured simultaneously and collected, on average, within 02:58 ± 01:08 s.d. (mm:ss) of entering their cage. The attending adult was typically noted at the time of collection. Subjects were anesthetized immediately using isoflurane until unresponsive and then decapitated. Brain, pituitary, and gonadal tissues were collected for a concurrent study. We collected trunk blood from birds in non-heparinized 5mL centrifuge tubes, which was put on ice. Following return to the lab, we centrifuged blood samples at 4°C for 10min in order to extract the plasma. Plasma was then stored at -80°C until hormone assays were performed.

### Endocrine Characterization

Our goal was to characterize circulating hormones throughout reproduction and parental care in rock doves. A schematic of the sampling points can be found in **Figure 1**. Briefly, we sampled individual males and females at nine time points over the course of reproduction. Individuals were only sampled once during this study; thus, datapoints within each hormone and sex are independent. Our groups consisted of 1) non-breeding, sexually mature birds, 2) nest-building, when birds were collected if at least one individual in the pair was observed carrying nesting material or shaping a nest, 3) laying, the morning the first egg was laid, 4) at clutch completion (i.e., the morning the second egg was laid), 5) mid-incubation (day 9), 6) late incubation (day 17), 7) hatching, the morning that the first nestling hatched, 8) early chick-rearing (first hatching nestling=5 days old), and 9) mid chick-rearing (nestling(s)=9 days old), which coincided with the average day that brooding ended and when nestlings were primarily fed a regurgitant instead of crop milk. Sample sizes for each hormone by sex and treatment can be found in **Supplemental Table 1**.

### Experimental Manipulation of Reproduction

We manipulated additional pairs to understand how changes to the nest environment influenced circulating hormone levels (**Figure 1**) which were compared to hormone levels from unmanipulated control birds at the same breeding time point (see *Reproduction Sampling Stages*). This approach also allowed us to investigate whether hormone levels were externally (i.e., presence or absence of nest contents) or internally (biological clock) driven, or if it was some combination of both. We accomplished this by comparing the endocrine response of these 5 hormones to similar manipulations based on reproductive stage (sampling point). As such, we removed the nest contents (eggs or nestlings) at multiple points during incubation (clutch completion, mid-incubation, late incubation) or at hatching. Nest content removal occurred the day before (~24hrs) collection. The goal of this manipulation was to simulate nest loss as it occurs following a predation event. We also added a nestling at mid-incubation (d8; simultaneously removing the pairs’ eggs) and allowed pairs to care for it for ~24 hours. This treatment effectively reduced the length of the incubation period. Finally, we prolonged incubation by 3 days (incubation period = 21d) by replacing viable eggs with fake or infertile eggs (collections occurred on d21) and extended hatching by prolonging incubation then adding ≥ 1 chick for ~ 24 hours before collecting the pair. This treatment group was meant to simulate the effect of lengthening incubation of viable eggs.

### Hormone Assays

We quantified hormone concentration (ng/mL) using a radioimmunoassay as previously described in detail by (38).

#### Corticosterone (CORT)

Briefly, we measured 50 µL of plasma and combined with 2,000 CPM of tritiated corticosterone to determine percent-recoveries. Next, we added 4 mL of freshly redistilled dichloromethane to each sample to extract the steroids from the plasma over a 3 h period. Extracts were dried under a stream of nitrogen in a water bath heated to 35°C and reconstituted using 550 µL of phosphate-buffered saline with gelatin (PBSG). We added a 100 µL aliquot to a scintillation vial and combined with 3mL scintillation fluid, which we used to determine percent recoveries. We assayed duplicate 200 µL aliquots by adding 100 µL (~10^4^ CPM) of tritiated label (Perkin Elmer NET399250UC) and 100 µL of antibody (MP Biomedical antibodies: 07-120016. Lot 3R3-PB-20F). Unbound steroid was stripped from solution by the addition of 500 µL of dextran coated charcoal followed by centrifugation at 3000 RPM. The supernatant was decanted into a scintillation vial, combined it with 3.5 mL scintillation fluid (Perkin Elmer Ultima Gold: 6013329), and counted for 6 minutes or within 2% accuracy on a Beckman 6500 liquid scintillation counter. Final hormone values were corrected using the individual recovery and volume correction for each sample. Mean recoveries were 87.61% and intra-(calculated using C.V. between duplicates) ranged from 3.31 - 8.83 and inter-assay variations was 5.19 (s.d. = 2.19). Assay detection limit was 11.06 ± 3.75 pg/tube.

#### Progesterone, Estradiol and Testosterone

We used a similar approach for the remaining hormones with some notable differences. First, we used 300µL of plasma for progesterone (200µL in laying females), 190-200µL for estradiol, and 175µL (100µL in late incubation and nestling sampling stages) for testosterone. Samples were extracted using 4mL of diethyl ether (Sigma Aldrich) over a 60-minute period. Plasma volumes were selected and validated to be between 30-70% bound for each hormone prior to beginning the assays. Doves generally have low circulating hormone levels requiring higher plasma volumes than in other species. Certain time points where known levels of a particular hormone were higher required less plasma volume for detection. Adjustments were made in the calculations of hormone concentrations based on plasma volumes as is typical for RIAs. We used tritiated label and antibody specific to each hormone (progesterone: label= Perkins-Elmer NET 38125OUC, antibody= Fitzgerald 2-Pr20T, 17βestradiol: label= Perkins-Elmer NET 51725OUC, antibody=Antibodies-online ABIN1826595 and [1,2,6,7,16,17-3H(N)] testosterone: label= Perkins-Elmer NET553250UC, antibody=Fitzgerald Industries, 20R-TR018w). We only conducted estradiol assays on females and testosterone in males because circulating levels tend to be very low in opposite sex.

Mean recoveries for progesterone were 61.91% and mean inter-assay variations was 12.19 (s.d. 8.41). The detection limit of the assays was 9.48 ± 5.76 pg/tube. Mean recoveries for estradiol were 71.61% (s.d 7.57). Inter-assay variation was 14.00 (s.d. 3.82). The estradiol assay detection limit was 6.11 ± 5.65 pg/tube. Intra-assay variation was generally high for estradiol owing to low circulating concentrations (e.g., differences of replicates of 0.06 ng/mL (replicate 1: 0.06 and replicate 2: 0.12) result in a relatively high C.V. of 30.86). Mean recoveries for testosterone were 84.29% (s.d. 9.30) and inter-assay variation was 5.87 (s.d. 2.81). The detection limit of the testosterone assay was 2.24 ± 0.92 pg/tube.

#### Prolactin

Because of the proteic nature of prolactin, plasma levels were determined by using a heterologous radioimmunoassay at the CEBC-CNRS (Chizé, France) as detailed in (39). For validation of the prolactin assay for rock doves, pooled plasma samples produced a dose-response curve that paralleled chicken prolactin standard curves (40). All samples were run in 2 assays and the intra- and inter-assay CVs were 9.58 and 11.83%, respectively.

## Analysis

We used general linear models to compare corticosterone, progesterone, and prolactin to sex, stage/treatment and their interaction term (PROC MIXED, SAS v. 9.4). If sex differences were present in circulating levels of CORT, progesterone, and prolactin, as indicated by a significant interaction term, then separate analyses for each sex were required. It was necessary to run these as separate models because the significant interaction term indicates a magnitude difference in the responsiveness of the hormone by sex. Because estradiol and testosterone were not assayed in both sexes, we conducted sex-specific general linear models of each circulating sex steroid as above excluding the sex term. We included all characterization (*hereafter*, sampling stage) or experimental manipulation groups (*hereafter*, treatment) in each analysis. We then compared differences between a set of groups using contrasts. We chose this targeted approach because we had a set of *a priori* hypotheses that we wanted to test (**Figure 2**). To improve the distribution of the dataset, we natural log-transformed all hormone variables.

We conducted pairwise comparisons to characterize changes in circulating hormone levels from non-breeding to breeding across all other sampling points. Next, we compared sampling stage groups to the adjacent or similar sampling points (nest-building *vs*. laying, laying *vs*. clutch completion, clutch completion *vs*. mid-incubation, mid-incubation *vs*. late incubation, clutch completion *vs*. late incubation, late incubation *vs*. hatching, hatching *vs*. nestling d5, nestling d5 *vs*. nestling d9). The goal was to determine how hormones changed over reproduction, specifically, across transition points (e.g., late incubation *vs*. hatching) and among similar behaviors (e.g., early, mid and late incubation). We then investigated how manipulating the nest contents affected hormone levels. We compared each manipulation to their respective control sampling groups (see **Figure 1**). These comparisons included removal of eggs at: clutch completion, mid-incubation, late incubation, and hatch. We also manipulated the length of the caretaking period and compared those treatments to a sampling stage control group. We decreased incubation length (added chicks at incubation d8), prolonged incubation (3 days after expected hatch day), and extended hatching (prolong plus chick(s) for a day) and compared them to controls at mid-incubation, late incubation or hatch, and hatch, respectively. Finally, to assess if biological schedule when the treatments occurred influenced circulating hormone levels, we compared the experimental manipulation treatment groups amongst themselves. These comparisons included: 1) removal of eggs at clutch completion *vs*. mid or late incubation, 2) removal of eggs at mid *vs*. late incubation, 3) removal of eggs at late incubation *vs*. hatching, and 4) prolonged incubation *vs*. extended hatching. A list of the pairwise comparisons for all sampling points and treatment groups can be found in **Tables 1** and **2**, respectively.

**Table 1:** Contrasts of pairwise sampling stage group comparison.

Contrasts	Corticosterone (*Males & Females*)	Progesterone (*Males & Females*)	Estradiol (*Females*)	Testosterone (*Males*)	Prolactin	
					*Males*	*Females*
Compared to non-breeding						
non-breeding *vs*. nest-building	ns	0.32 ± 0.13 *p*=0.013	-0.53 ± 0.14 *p<*0.001	ns	ns	0.49 ± 0.13 *p*<0.001
non-breeding *vs*. laying	-0.15 ± 0.09 *p*=0.097	ns	-0.54 ± 0.14 *p*<0.001	ns	-0.25 ± 0.15 *p*=0.100	0.60 ± 0.13 *p*<0.001
non-breeding *vs*. clutch completion	-0.31 ± 0.09 *p*<0.001	ns	-0.57 ± 0.14 *p*<0.001	0.37 ± 0.22 *p*=0.085	ns	ns
non-breeding *vs*. mid-incubation	ns	0.31 ± 0.12 *p*=0.009	-0.25 ± 0.14 *p*=0.074	ns	-0.46 ± 0.14 *p*=0.001	ns
non-breeding *vs*. late incubation	-0.31 ± 0.09 *p*<0.001	0.37 ± 0.13 *p*=0.006	-0.43 ± 0.14 *p*=0.003	ns	-0.89 ± 0.14 *p*<0.001	-0.37 ± 0.13 *p*=0.006
non-breeding *vs* hatching	-0.31 ± 0.09 *p*<0.001	0.42 ± 0.15 *p*=0.006	-0.55 ± 0.15 *p*<0.001	ns	-1.12 ± 0.15 *p*<0.001	-0.50 ± 0.13 *p*<0.001
non-breeding *vs*. nestling d5	-0.40 ± 0.09 *p*<0.001	ns	-0.42 ± 0.14 *p*=0.004	0.42 ± 0.22 *p*=0.059	-0.99 ± 0.15 *p*<0.001	-0.37 ± 0.13 *p*=0.005
non-breeding *vs*. nestling d9	ns	0.33 ± 0.14 *p*=0.015	-0.86 ± 0.15 *p*<0.001	-0.46 ± 0.22 *p*=0.039	-0.73 ± 0.15 *p*<0.001	-0.23 ± 0.13 *p*=0.087
Compared to adjacent time points						
nest-building *vs*. laying	ns	-0.51 ± 0.14 *p*<0.001	ns	ns	-0.31 ± 0.16 *p*=0.058	ns
laying *vs*. clutch completion	-0.27 ± 0.09 *p*=0.065	0.32 ± 0.15 *p*=0.031	ns	ns	ns	-0.50 ± 0.13 *p*<0.001
clutch completion *vs*. mid-incubation	0.18 ± 0.09 *p*=0.041	ns	0.33 ± 0.14 *p*=0.021	ns	-0.31 ± 0.15 *p*=0.044	ns
clutch completion *vs*. late incubation	ns	ns	ns	-0.44 ± 0.23 *p*=0.059	-0.74 ± 0.15 *p*<0.001	-0.47 ± 0.13 *p*<0.001
mid-incubation *vs*. late incubation	-0.17 ± 0.08 *p*=0.043	ns	ns	ns	-0.43 ± 0.15 *p*=0.004	-0.52 ± 0.13 *p*<0.001
late incubation *vs*. hatching	ns	ns	ns	ns	ns	ns
hatching *vs*. nestling d5	ns	-0.46 ± 0.17 *p*=0.006	ns	ns	ns	ns
nestling d5 *vs*. nestling d9	0.29 ± 0.09 *p*=0.002	0.37 ± 0.15 *p*=0.016	-0.44 ± 0.15 *p*=0.004	-0.89 ± 0.24 *p*<0.001	ns	ns

Statistically significant results are included (estimates ± SE and p-values; ‘ns’, not significant).

The direction of the difference is depicted as either pink (an increase in hormone concentration between groups) or blue (a decrease). Lighter colors indicate suggestive, but not significant group differences (0.05 < p < 0.1).

**Table 2:** Contrasts of pairwise treatment group comparison.

Contrasts	Corticosterone (*Males & Females*)	Progesterone (*Males & Females*)	Estradiol (*Females*)	Testosterone (*Males*)	Prolactin	
					*Males*	*Females*
Comparisons to controls						
clutch completion *vs*. remove eggs at clutch completion	ns	ns	0.68 ± 0.15 *p*<0.001	-0.52 ± 0.23 *p*=0.026	ns	0.25 ± 0.13 *p*=0.068
mid-incubation *vs*. add nestlings at mid-incubation	-0.16 ± 0.09 *p*=0.079	ns	0.49 ± 0.16 *p*=0.002	ns	ns	ns
mid-incubation *vs*. remove eggs at mid-incubation	0.18 ± 0.09 *p*=0.038	ns	ns	-0.85 ± 0.23 *p*<0.001	0.29 ± 0.16 *p*=0.068	ns
late incubation *vs*. remove eggs at late incubation	0.23 ± 0.09 *p*=0.012	ns	ns	-0.52 ± 0.24 *p*=0.036	0.52 ± 0.16 *p*=0.001	0.53 ± 0.13 *p*<0.001
late incubation *vs*. prolong incubation	ns	ns	0.56 ± 0.15 *p*<0.001	ns	ns	ns
hatching *vs*. add nestlings at mid-incubation	ns	ns	0.79 ± 0.17 *p*<0.001	ns	0.87 ± 0.16 *p*<0.001	0.68 ± 0.14 *p*<0.001
hatching *vs*. remove nestlings at hatch	ns	ns	0.40 ± 0.16 *p*=0.014	-0.89 ± 0.24 *p*<0.001	0.81 ± 0.16 *p*<0.001	0.56 ± 0.13 *p*<0.001
hatching *vs*. extend hatching	ns	ns	0.72 ± 0.16 *p*<0.001	ns	ns	ns
hatching *vs*. prolong incubation	ns	ns	0.69 ± 0.16 *p*<0.001	-0.45 ± 0.24 *p*=0.058	0.32 ± 0.16 *p*=0.047	ns
Within manipulation comparisons						
prolong incubation *vs*. extend hatching	-0.16 ± 0.09 *p*=0.079	ns	ns	ns	ns	ns
remove eggs at clutch completion *vs* at mid-incubation	0.40 ± 0.09 *p*<0.001	ns	ns	-0.54 ± 0.24 *p*=0.022	ns	ns
remove eggs at clutch completion *vs*. at late incubation	0.27 ± 0.09 *p*=0.003	ns	-0.37 ± 0.15 *p*=0.013	-0.43 ± 0.24 *p*=0.076	-0.42 ± 0.16 *p*=0.011	ns
remove eggs at mid-incubation *vs*. at late incubation	ns	ns	ns	ns	ns	ns
remove eggs at late incubation *vs*. chicks at hatching	ns	ns	ns	ns	ns	ns

Statistically significant results are included (estimates ± SE and p-values; ‘ns’, not significant).

The direction of the difference is depicted as either pink (an increase in hormone concentration between groups) or blue (a decrease). Lighter colors indicate suggestive, but not significant group differences (0.05 < p < 0.1).

To explore how hormones were intercorrelated, we conducted a series of Pearson correlation coefficients [R 3.6; package ‘Hmisc, v. 4.4-1; (41)]. We also conducted variable selection using Boruta analyses, a Random Forest approach [R. 3.6; package ‘Boruta’; v. 7.0.0; (42)], on males and females. This approach highlights the most important attributes that significantly affected circulating hormones levels. It allowed us to determine if individual hormone were statistically important, and also generally, corroborated the simpler correlational analysis. For manipulation analyses, we combined similar treatment types. We created two groups: ‘removal’ (m.inc3, m.inc9, m.inc17, and m.hatch) and ‘duration change’ (m.inc8, prolong, and extend). This approach allowed us to compare which hormones co-varied by general treatment types.

Finally, to synthesize how circulating hormone levels co-varied by sampling stage, we conducted a PERMANOVA (R v. 3.6; package ‘vegan’ v. ﻿2.5-6) in males and females. To fill missing values, we used the sex- and stage-specific hormone averages. By filling missing values, it allowed us keep individuals in this analysis that would have been removed due to one missing hormone value. To remain transparent, we report the results with and without filling these missing values. To conduct the PERMANOVA, we square-root transformed all hormone values and then created a dissimilarity matrix using Bray-Curtis distances. We then compared co-variation across sampling stages. The goal of this analysis was to determine if hormones co-varied across sampling stages and to provide a visual of how sampling stages clustered for each sex.

## Results

**Figure 3:**
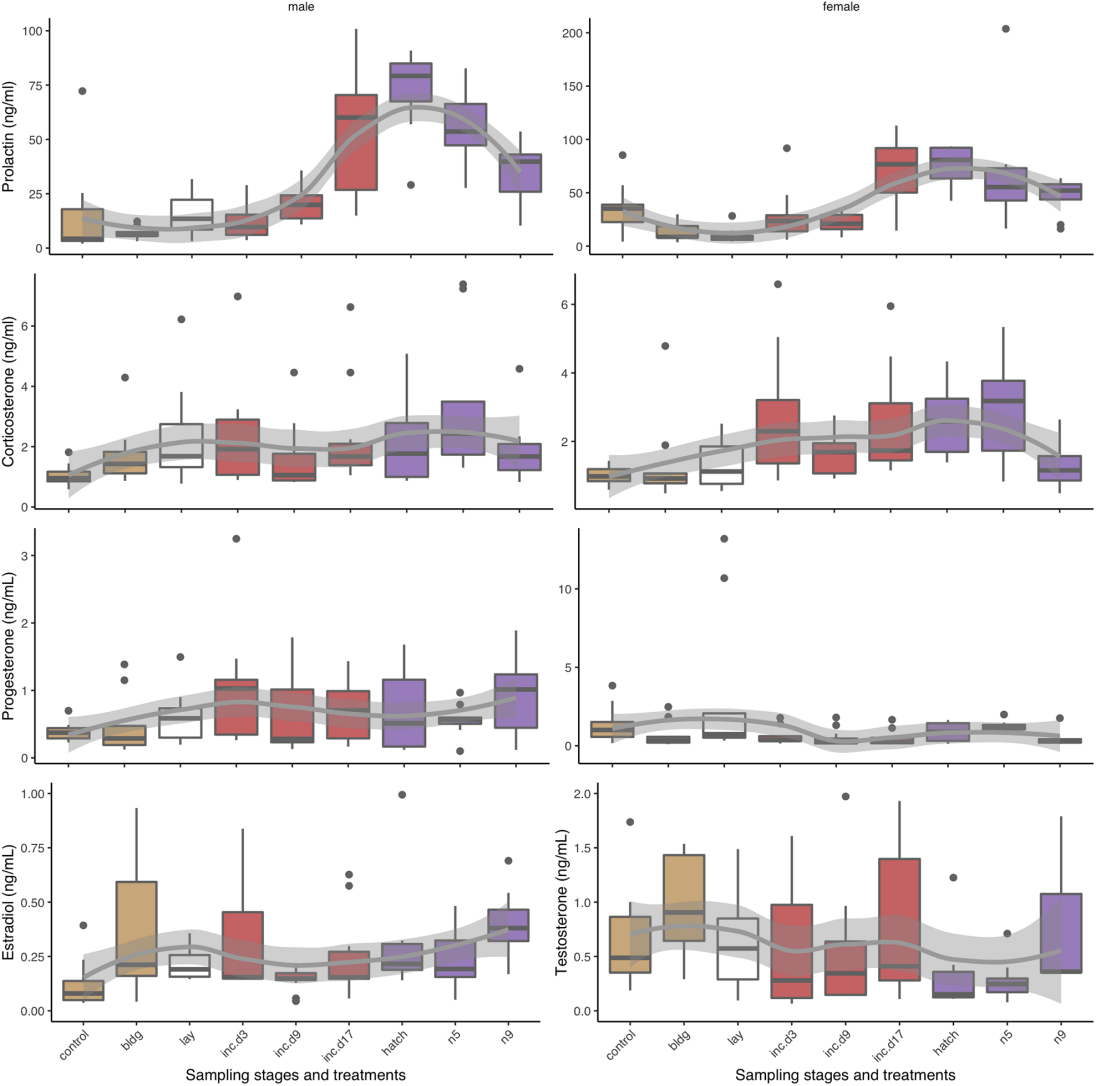
Box plots of circulating hormone levels (ng/mL) of reproduction sampling stages over parental care for corticosterone, progesterone, prolactin, estradiol, and testosterone in males (left) and females (right). Box colors indicate different sub-stages: non-breeding (control) and nest-building (bldg; brown), laying (lay; white), incubation (red, inc.d3=clutch completion, inc.d9=mid-incubation, and inc.d17=late incubation), and the nestling period (purple; hatch, n5=nestling d5, and n9=nestling d9). Boxes represent median and upper and lower quartiles. Whiskers represent the most extreme data point that are within 1.5 of the interquartile range.

Plots of CORT, progesterone, and prolactin for both sexes, and estradiol for females and testosterone for males across reproduction can be found in **Figure 3**. We did not include pair id in our models because it was not significantly related to any of the hormones. We also compared whether circulating hormones differed between attending and non-attending adults across stages and treatments, but we found no significant difference across all hormones.

### Sex Differences Between Sampling Stage Groups and in Response to Treatment

We found that CORT was significantly related to sampling stage/treatment but not sex or the interaction term (sampling stage/treatment: *F*_15, 293_ = 4.9, *p* < 0.001, sex: *F*_1, 293_ = 1.9, *p* = 0.172, sampling stage/treatment*sex *F*_15, 293_ = 1.3, *p* = 0.209). For progesterone, we found a suggestive though insignificant interaction effect (sampling stage/treatment: *F*_15, 178_ = 3.4, *p* < 0.001, sex: *F*_1, 178_ = 2.6, *p* = 0.111, sampling stage/treatment*sex *F*_15, 178_ = 1.7, *p* = 0.066), which indicated sex-specific difference in hormone levels by stage and treatment. For prolactin, we also found a significant interaction effect (sampling stage/treatment: *F*_15, 294_ = 25.5, *p* < 0.001, sex: *F*_1, 295_ = 28.0, *p* < 0.001, sampling stage/treatment*sex *F*_15, 294_ = 1.8, *p* = 0.034). Thus, for prolactin (but not CORT or progesterone), we assessed the effect of sampling point and treatment separately for each sex. We report the results of these sex-specific models below.

### Reproduction Sampling Stages

We compared each hormone to sampling stage to determine if there were differences in circulating hormone levels over the reproductive period. Results of pairwise comparisons of sampling stages for all hormones can be found in **Table 1**.

#### CORT

We found that when we compared non-breeding individuals, CORT was higher at clutch completion, late incubation, hatching, and nestling d5 (**Table 1** and **Figure 3**). There were no differences in CORT levels between non-breeding birds and those at nest-building, mid-incubation, or nestling d9. When we compared sampling stages with adjacent time points or where similar parental behavior was displayed, we found a significant increase in CORT between clutch completion and mid- and late incubation and a decrease between nestling d5 and nestling d9.

#### Progesterone

Models of sampling stage were significant (*F*_15, 194_ = 3.1, *p* < 0.001; **Table 1** and **Figure 3**). When progesterone levels were compared between non-breeding individuals and all other sampling stages, we found significantly lower levels of progesterone at nest-building, mid-incubation, late incubation, hatching and nestling d9, but much overlap and variation was present. Comparisons of adjacent breeding sampling stages indicated that progesterone was higher at laying than nest-building, was lower at clutch completion than laying, increased between hatching and nestling d5, and decreased between nestling d5 and nestling d9.

#### Estradiol

In females, circulating concentrations of estradiol significantly differed by sampling stage/treatment (*F*_15, 139_ = 9.0, *p* < 0.001; **Table 1** and **Figure 3**). Pairwise comparisons indicated that, estradiol was higher at nest-building, laying, clutch completion, hatching and nestling d9 compared to non-breeding females. Estradiol decreased between clutch completion and mid-incubation and increased between nestling d5 and d9.

#### Testosterone

Circulating testosterone in males differed by sampling stage/treatment (*F*_15, 138_ = 4.7, *p* < 0.001; **Table 1** and **Figure 3**). Compared to non-breeding males, testosterone was significantly higher when nestlings were 9 days old and increased significantly between nestling d5 and d9.

#### Prolactin

*Males:* Prolactin varied significantly across sampling stages in males (*F*_15, 146_ = 13.1, *p* < 0.001; **Table 1** and **Figure 3**). Prolactin increased between non-breeding and all sampling periods except at nest-building and clutch completion. Generally, prolactin increased over the course of the incubation period when adjacent stages were compared (clutch completion compared to mid and late incubation and mid-and late incubation), which was expected.

*Females:* Prolactin levels significantly varied across sampling stage in females (*F*_15, 148_ = 14.5, *p* < 0.001; **Table 1** and **Figure 3**). In our birds, prolactin levels were lower in non-breeding compared to nest-building and laying females. In females, prolactin increased from non-breeding compared to late incubation, hatching, and during the nestling period (d5). It also increased between laying and clutch completion, clutch completion and late incubation, and mid- and late incubation.

### Assessing Inter-Relatedness of Hormones

Across both sexes and all reproductive sampling stages (excluding non-breeding) CORT was weakly related (Spearman rho) to prolactin and progesterone (CORT vs. prolactin: *R* = 0.22, *p* = 0.004, CORT *vs*. progesterone *R* = 0.16, *p* = 0.038). Recall that testosterone and estradiol are sex-specific; thus, only progesterone, prolactin, and CORT could be compared in both sexes. All subsequent correlations are sex-specific. In females, we found a moderately strong positive correlation between prolactin and CORT (*R* = 0.41, *p* < 0.001) and a negative weak correlation between prolactin and progesterone (*R* = -0.28, *p* = 0.017). When we limited female sampling stages to those with eggs then there was a strong positive correlation between CORT and prolactin (*R* = 0.60, *p* < 0.001). During the nestling period, CORT and progesterone (*R* = 0.44, *p* = 0.016), estradiol and progesterone (*R* = -0.38, *p* = 0.036) were positively and negatively correlated, respectively. Positive correlations between prolactin and CORT were highest at clutch completion (*R* = 0.79, *p* = 0.006) and at hatching (*R* = 0.70, *p* = 0.035). In males, there were no significant correlations across breeding stage; however, when sampling stages were limited to the nestling period then prolactin and testosterone were significantly negatively correlated (*R* = -0.41, *p* = 0.025).

To determine if sampling stage or other hormones were important attributes, we conducted variable selection of each hormone by sex. In females, we found that for CORT, sampling stage and prolactin were important attributes while progesterone was tentatively important. For progesterone, sampling stage was important and CORT was tentatively important while for estradiol only sampling stage was important. For prolactin, we found that only corticosterone and sampling stage were attributes of importance. In males, only treatment was related to CORT, progesterone, testosterone and prolactin.

**Figure 4:**
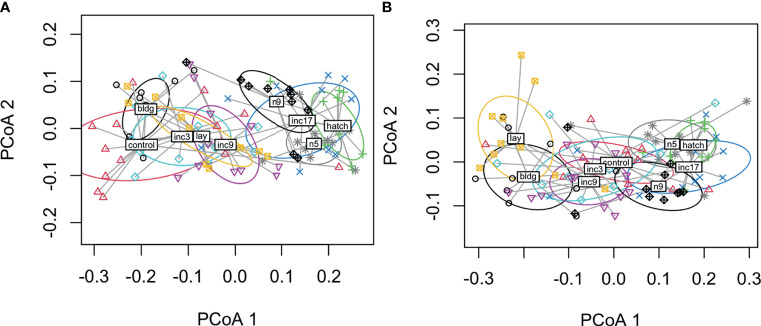
Dispersion plots of five hormones in male **(A)** and female **(B)** rock doves by sampling stage (control=non-breeding, bldg=nest building, lay=lay egg 1, inc3= clutch completion, inc9=mid-incubation, inc17= late incubation, hatch=hatching, n5=nestling day 5, and n9=nestling d9).

**Figure 5:**
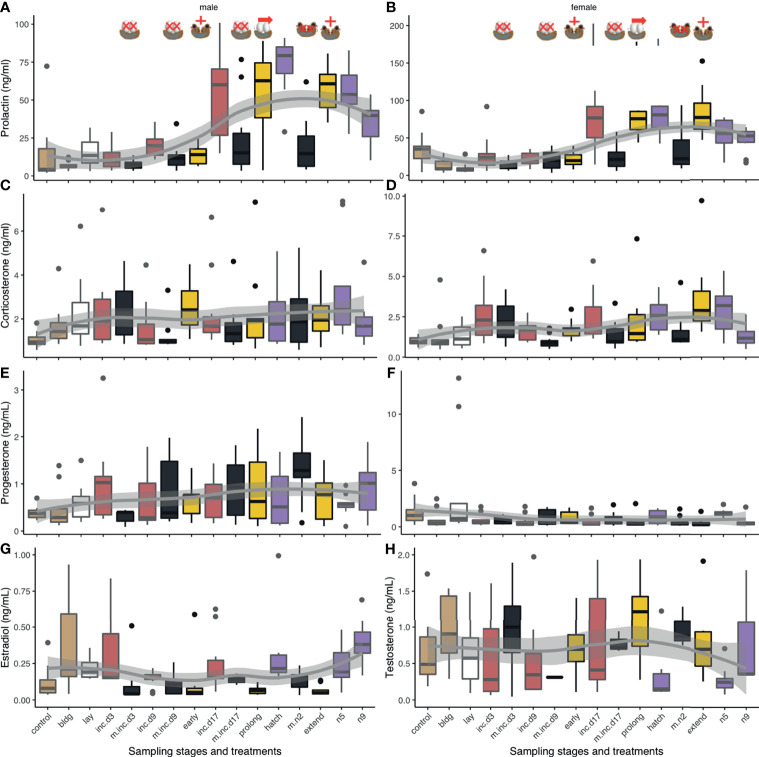
Boxplots of circulating hormone levels (ng/mL) of experimental manipulations (maize yellow) and their pairwise controls (green) for corticosterone **(A, B)**, progesterone **(C, D)**, prolactin **(E, F)**, and the sex steroids [testosterone **(G)**, estradiol **(H)**]. Non-breeding time points are included for reference. Controls (black text) are adjacent to manipulations (blue text) unless there are more than one experimental manipulation for a given time point. Eggs were removed at clutch completion (cc), mid-incubation (mi), and late incubation (li). Chicks were added at mid-incubation and removed at hatch. The incubation period was prolonged by 3 days (prolong) prior to adding chicks for 1d (extend). Box colors indicate different sub-stages periods: non-breeding (control) and nest-building (bldg; brown), laying (lay; white), incubation (red, inc.d3=clutch completion, inc.d9=mid-incubation, and inc.d17=late incubation), and the nestling period (purple; hatch, n5=nestling d5, and n9=nestling d9). Contents for a given manipulation are indicated by the presence of eggs or chicks in the nest and were either removed (black and X), added (yellow; +), or the natural duration of incubation was prolonged (yellow; →). Boxes represent median and upper and lower quartiles. Whiskers represent the most extreme data points that are within 1.5 of the interquartile range.

When we compared all hormones by sampling stage using PERMANOVA, we found significant differences in males (*F*_8, 70_ = 9.8, *p* < 0.001, *R* ^2^ = 0.53; no missing values *F*_8, 86_ = 13.0, *p* < 0.001, *R* ^2^ = 0.55) and females (*F*_8, 74_ = 10.0, *p* < 0.001, *R* ^2^ = 0.52; no missing values: *F*_8, 87_ = 11.1, *p* < 0001, *R* ^2^ = 0.50). The plots indicate that hormones cluster by early and late sampling stages with early stages consisting of non-breeding, nest-building, laying, clutch completion, mid-incubation and late stages including late incubation, hatching, nestling d5 and d9 (**Figure 4**). In males, the nest-building and non-breeding control groups overlapped with the early sampling stages, but the centroids were somewhat removed. Females show more overlap across early and late stages with nest-building and laying being reasonably distinct clusters (**Figures 5A, B**).

### Experimental Manipulation of Reproduction

We compared treatment groups to their respective control sampling stages and to other similar treatment groups. All results from these manipulations are presented in **Table 2** and **Figure 5**.

#### CORT

When we compared CORT levels following experimental manipulations to their control groups, we found that egg removal at mid- and late incubation decreased CORT (**Table 2** and **Figures 5A, B**). To determine if there were differences in hormonal response to experimental manipulation that might be explained by the stage of breeding in which treatment occurred (or similarly, by the change in cue), we compared experimental manipulations to similar treatments. When we compared treatments where eggs were removed at clutch completion compared to at mid- or late incubation, we found that CORT levels decreased at later breeding stages.

#### Progesterone

We did not find any significant differences in circulating progesterone when experimental treatments were compared to their breeding stage-specific controls or between similar types of manipulations suggesting that manipulating the nest contents did not significantly affect circulating progesterone (**Table 2** and **Figures 5C, D**).

#### Estradiol

In response to nest loss, female estradiol levels decreased when eggs were removed at clutch completion or when nestlings were removed at hatching (**Table 2** and **Figure 5H**). Circulating estradiol also decreased when incubation duration was prolonged or decreased, or hatching was extended. When we compared treatments amongst each other, we found that removing eggs at late incubation resulted in higher levels of estradiol than at clutch completion.

#### Testosterone

Male testosterone levels increased after nest contents were removed at clutch completion, mid-incubation, late incubation, and at hatching (**Table 2** and **Figure 5G**). There were no other differences in testosterone among treatments related to manipulating incubation or hatching length (i.e., prolong, extend, or decrease treatment groups). The timing of reproduction of a given treatment did influence circulating testosterone levels. Testosterone was significantly higher when eggs were removed at mid-incubation or late incubation compared to clutch completion.

#### Prolactin

*Males:* Prolactin decreased following removal of eggs at late incubation compared to its control group (**Table 2** and **Figure 5E**). When nestlings were removed near hatch, prolactin levels significantly decreased. There were several differences between sampling stages and treatment groups that can likely be attributed to normal variation in prolactin over the course of breeding. Prolonging incubation by 3 days showed a decrease in prolactin as compared to hatching but adding chicks after this prolongation had no effect on prolactin in males. Given the lack of difference between prolong and extend treatment groups and the extend treatment group and its control sampling stage (hatch), we cannot rule out that this decrease is merely a naturally occurring decrease in prolactin that is unrelated to treatment. This result may lend support to the internal timing hypothesis. Circulating prolactin was lower between the hatching sampling stage and when the incubation period was experimentally decreased (chicks added at mid-incubation); however, this significant difference can be attributed to a natural increase in prolactin between mid-incubation and hatching because this treatment group did not differ from its mid-incubation control. Similarly, the only difference between male prolactin levels across treatment groups occurred when removal of eggs at clutch completion *vs*. late incubation were compared, which can be attributed to normal increases in circulating levels at the end of incubation and beginning of the nestling period.

*Females:* Prolactin levels in females were lower at late incubation compared to their respective controls (**Table 2** and **Figure 5F**). When chicks were removed at hatching, circulating prolactin also decreased. Like males, females had lower prolactin levels when the incubation period was decreased by adding chicks at mid-incubation, which as before, can likely be attributed to reproductive timing and do not represent a treatment effect. There were no significant differences when similar treatment groups were compared; thus, prolactin response to nest content loss was similar across the incubation and the nestling period, or when incubation was prolonged as compared to when the hatching period was extended.

### Assessing Inter-Relatedness of Hormones

When we assessed correlations and relatedness of these hormones in removal and duration manipulation groups in each sex, we found there were no correlations and only a few important hormone associations found using Boruta including: 1) males from remove groups where there was a tentative relationship between testosterone and progesterone, 2) males from change groups where prolactin was important for progesterone (though there was only tentative support of this association when prolactin and not progesterone was the response variable), 3) female remove groups where CORT was important for progesterone (but not vice versa), and 4) female period change groups for prolactin where an association between prolactin and progesterone was found (both directions).

**Figure 6:**
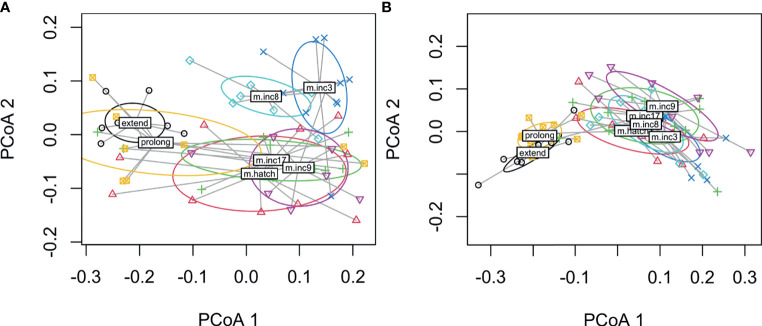
Dispersion plots of five hormones in male **(A)** and female **(B)** rock doves by experimental manipulation treatment (m.inc3 = remove eggs at clutch completion, m.inc9 = remove eggs at mid-incubation, m.inc17 = remove eggs at late incubation, m.hatch = remove nestlings at hatching, m.inc8 = reduce incubation period to 9d, prolong = prolong incubation to 21d and extend = extend length of time to hatch).

When we compared all hormones across experimental manipulations using PERMANOVA, we found significant differences among treatments in both males (*F*_6, 61_ = 7.0, *p* < 0.001) and females (*F*_6, 62_ = 11.5, *p* < 0.001). Plots indicate that hormones cluster by removal and parenting time periods though decreasing incubation period clustered with the removal treatments in both males and females (**Figures 6A, B**). In males, decreasing incubation period clustered loosely with removing eggs at clutch completion. These results suggest a general pattern in hormonal response to external stimuli, timing may limit hormonal response when the incubation period length is decreased, and at least in males, the removal of eggs at clutch completion and decreasing the incubation period length treatments seem to result in a similar overall hormone response.

## Discussion

We sought to characterize the role of reproductive hormones in males and females over the course of the reproductive stages in a biparental species. To extend the utility of the study, we also conducted a series of experimental manipulation treatments to determine if circulating hormones were influenced by external stimuli alone or if a biological schedule influenced (e.g., attenuated) this response. We found clear differences across sampling stages and in response to experimental treatments in all hormones sampled. Across all five hormones, we found similarities in earlier (up to mid-incubation) compared to later (from late incubation through nestling d9) sampling stages in males and females. This clustering coincides with major behavioral differences in birds, i.e., moving from mating behavior to egg-laying and the onset and sustainment of incubation compared to late incubation and chick-rearing behaviors. Females exhibited more overlap which may indicate that these hormones vary in a more continuous manner than males. However, hormone levels were not related to a binary variable describing the presence or absence in the nest of pairs at collection thereby suggesting sampling stage and not immediate behavior was more significantly related to hormone concentrations. Overall, our data support sex-specific response differences to nest content loss and to changes in the length of the incubation period. Females generally reduced circulating sex steroids in response to manipulation whereas males increased them.

### Reproduction Sampling Stages

#### CORT

Our data show that CORT increased during late incubation through the mid-nestling period (d5) compared to non-breeding individuals. A similar pattern in circulating CORT was observed in other dove species (24, 43–45) and coincides with feeding rate and parental metabolism (24). Increased CORT during late incubation and early chick-rearing may indicate an increase in metabolic rate or overall metabolism. CORT, a metabolic hormone, typically rises during breeding in birds, as a result of increased parental effort (24, 46). One interesting difference between doves and many other bird species is that dove parents produce crop milk. Crop milk allows Columbid nestlings to grow at one of the fastest rates among birds (47). To meet the needs of their growing nestlings, and perhaps the increased energetic cost of producing crop milk, parents double their food consumption, but it is insufficient to maintain body mass (43, 44). Adult mass declines over the nestling period, which suggests a negative energy state (44). Among the individuals used in this study, we observed vascularized crops and crop milk at late incubation, hatching and nestling d5 (SHA personal observation). By day 9 of the nestling period, parents fed their nestlings primarily a regurgitant of seeds (35), which coincides with a decrease in basal CORT.

Both CORT and prolactin promote parental hyperphagia in doves, which is linked to the negative energy state observed during chick-rearing (7, 24, 26, 44). Non-breeding doves treated with intracerebroventricular (ICV) injections of dexamethasone (a synthetic glucocorticoid) increased their food intake (44). However, intramuscular injections of dexamethasone did not increase food consumption (44, 48), which suggests that high systemic circulating levels of CORT are needed to stimulate hyperphagia in doves (44). P-glycoprotein in the blood brain barrier actively pumps dexamethasone out of the brain (49). This effect has been well-documented in mammals (50). The lack of hyperphagic response could be an artifact of low dexamethasone in the brain; thus, hyperphagia induced by CORT would be blocked. Hyperphagia has also been blocked using ICV injections of the glucocorticoid antagonist, RU486, which further supports the role of glucocorticoids in promoting increased energy consumption during parenting (44). While CORT has been implicated in hyperphagia of breeding and migratory birds, prolactin has also been connected to this behavior in breeding Columbids (51–54).

#### Prolactin

Prolactin, a critical hormone in avian reproduction that is associated with parental care, gonadotrophic inhibition, and crop milk production, increases during late incubation, peaks several days after hatching and continues to decline for the remainder of the nestling period in pigeons. This pattern is probably associated with crop milk production in pigeons, but in other species, is associated with incubation and caregiving (17, 18, 25). There is evidence that CORT has an effect (sometimes strong) on prolactin (17, 26). As with CORT, ICV injections of prolactin increased food consumption in breeding and non-breeding doves (53, 54); however, prolactin does not seem to regulate hyperphagia in migratory birds (55). Prolactin-induced CORT synthesis [as a result of ICV injections of prolactin (26)] and hyperphagia may induce hypothalamic expression of neuropeptide Y (56), which is associated with increased food consumption. Over the nestling period, food intake of parents continues to increase yet circulating CORT and prolactin decrease (22, 43, 44). This decline in prolactin in both male and female doves coincides with a decline in crop milk production. There is no known data on the metabolic rate of rock dove parents during crop milk production; thus, experimental research is needed to separate the role of CORT and prolactin on parental hyperphagia and metabolism in Columbids.

#### Progesterone

Both progesterone and estradiol promote an LH surge prior to ovulation (14). Progesterone, specifically, induces muscular contractions of the oviduct in females during oviposition (6). We did not find significant sex-specific differences in progesterone levels over incubation; however, this lack of significance is likely due to the low concentrations of circulating progesterone at all except a few sampling stages and its generally high variance at these stages. Progesterone levels, as anticipated, were low for most of the sampling period. Circulating levels peaked during early breeding and were maintained at low or undetectable levels throughout the incubation and nestling periods. We did not detect an increase in progesterone when the second egg was laid, However, progesterone increases several hours prior to oviposition and is only detectable for a short period after the egg is laid (57); thus, we may have simply missed the window at clutch completion when this rise was detectable, though ring-necked doves also failed to show high progesterone levels after the second egg was laid (11). We did find an increase in progesterone at nestling d5, which decreased by nestling d9. This result, in conjunction with the observed increase in sex steroids at this sampling stage, may indicate a return to breeding readiness in preparation for a new breeding attempt. Overlapping nesting attempts were common in this colony (SHA personal observation). Silver (11) and Askew et al. (10) also found low circulating progesterone (in males) throughout the breeding cycle. Instead, variation in the progesterone receptor (and the binding of progesterone to this receptor) in the hypothalamus, not circulating progesterone, may contribute to behavioral changes, particularly in males where it has been associated with the onset of incubation (10).

#### Testosterone and Estradiol

We found that circulating estradiol increased in females during the laying period (laying and clutch completion). Estradiol then declined during incubation and increased by nestling d9. This rise in estradiol on nestling d9 and an observed increase in follicle size (unpublished data) suggests that females were preparing to re-nest. In males, circulating testosterone was low throughout breeding, which is typical (11). As with female estradiol, males significantly increased circulating testosterone at nestling d9. Because males respond to female reproductive behavior as opposed to breeding stage (29), an increase in testosterone in the middle of the nestling period suggests that females exhibited behaviors indicative of receptivity to mate. The concurrent rise in sex steroids of males and females suggests that these pairs were preparing both behaviorally and physiologically to re-nest, which is normal in this population.

### Inter-Relatedness of Hormones

When we assessed how hormones co-varied, we generally found that CORT, prolactin, and progesterone were related across sampling stages in females. The positive relationship between CORT and prolactin (24) and CORT and progesterone (58) is well-established. We did not find that estradiol and progesterone were related, as expected, but as discussed above, we likely missed the window during laying where these hormones peaked. In males, we did not find any covariation in these four hormones across all sampling stages; however, there was a significant negative relationship between prolactin and testosterone during the nestling period. This may be related to the well-known inhibitory effect of prolactin on the GnRH/LH/FSH pathway (59). When we used a multivariate approach, we found that both sexes showed significant clustering of sampling stages in hormone space. Specifically, sampling stages tended to cluster into early and late reproductive stages. Females showed more overlap across early and late breeding stages with nest-building and laying being somewhat distinct clusters. In males, non-breeding and nest-building centroids were more closely related among the early stages than those with eggs (though much overlap was present, particularly with the non-breeding group). Given the vast differences between hormones and physiology at these time points, these results were expected.

### Experimental Manipulation of Reproduction

*Hormone Response Following Nest Loss:* The response to nest content loss (either egg or nestling) typically caused adults to end their attendance of the nest (88-100% of all adults across all stages) and circulating levels differed by hormone and stage. CORT decreased following nest loss during mid and late incubation. Prolactin decreased in males when nest loss occurred at late incubation or at hatch. Females had a similar decrease in prolactin following nest loss at late incubation and hatching, which suggests that both sexes respond to the external stimuli of eggs or chicks to maintain increased prolactin levels that are necessary for crop milk production. In females, estradiol decreased to approximately non-breeding levels when nest contents were removed at clutch completion and at hatching while, in males, circulating testosterone increased following any nest loss. Generally, prolactin, estradiol and testosterone were more responsive to nest content loss than CORT or progesterone. Increased estradiol and testosterone have previously been observed following nest loss (28, 60). Because increased circulating estradiol in females and testosterone in males suggest gonadotroph induction, steroids can be used to roughly infer reproductive function. Our data suggest that, at least initially, females decrease their reproductive function whereas males ramp theirs up. It is not unexpected that female sex steroid hormonal response to nest content loss is slower than males. The short delay of 1 day between nest loss and sampling is insufficient for females to initiate final rapid follicle development leading to egg-laying and begin preparing to produce a replacement clutch. Increased testosterone has been observed after nest loss in other species [reviewed in (60)]. One hypothesis that could explain why males might increase testosterone quickly after nest loss (despite their mate reducing their reproductive function) is that by increasing testosterone and concurrent courtship behaviors males could stimulate females to return to breeding condition faster than they otherwise might (61, 62). Exposure to courting males can stimulate marked gonadal growth and gonadotrophic hormone secretion in females (61, 62). Quickly re-nesting following nest loss would be selectively favored in a species with a fast pace of life that incurs high nest loss, as is common among open-cup nesting birds like the rock dove. Studies have suggested that increased testosterone only occurs after nest loss and not following a normal re-nesting (multiple brooding) attempt following a successful nesting attempt (60). However, the increase in testosterone at day 9 of the nestling period may suggest rock doves increase testosterone prior to normal re-nesting thereby suggesting a species and/or a mating system distinction. More work is needed to confirm these results.

*Comparison of Timing of Hormone Response to Nest Loss:* To determine whether differences in the physiological response to nest loss was based on breeding sub-stage (i.e., biological schedule), we compared removal treatments across the breeding schedule. We found that CORT was higher when nest contents were lost at clutch completion compared to mid or late incubation. We observed higher male prolactin levels when nest loss occurred at late incubation compared to clutch completion, but we attribute this to naturally higher levels at this time. We also found that estradiol was lower after nest content loss at clutch completion compared to late incubation while testosterone was lower at clutch completion than at mid-incubation. This result may suggest that there is an attenuated response in males to nest loss during the laying period. It is unclear what mechanism underlies these differences in hormonal responses at different times during breeding, but it is likely related to physiological differences of individuals at different points during reproduction and/or responsiveness of the neuroendocrine system to the perturbation.

*Manipulating incubation and hatching period length:* When the length of incubation was decreased, CORT increased in both sexes while estradiol in females decreased. Birds were observed attending these nests (88% of females and 22% of males with normally one parent attending the nest at a time). When we prolonged incubation or extended hatching, estradiol also decreased. Prolonging incubation indicated a decrease in male, but not female, circulating prolactin compared to its hatching control group. Thus, manipulating the length of incubation or hatching had a similar effect on circulating estradiol, but varied in the other hormones. Bird incubation and brooding behavior also was prolonged as adults were observed attending these nests in all documented cases. Females were typically found on the nest at collection though this could be a timing issue related to attendance shifts in this species (90% of females and 10-11% of males). Manipulating the length of incubation, either by decreasing or increasing it, or extending hatching was predicted to have little effect on sex steroids because circulating levels remain low during parental care. Our treatments decreased estradiol below what was expected at this sampling stage which we cannot explain. The increase in CORT when nestlings were added during mid-incubation could be related to increased metabolism needed to feed nestlings Adults at mid-incubation do not produce crop milk. Parents from this treatment group did, however, attempt to feed their foster nestlings: we observed parents caring for young and when parents were sampled, the crops of these nestlings were filled with a clear liquid (personal observation). Thus, parents attempted to feed these chicks, but were unable to do so adequately due to a lack of crop milk.

### Inter-Relatedness of Hormones Following Experimental Manipulation

When we compared hormones of experimental manipulations using a multivariate approach, we found that treatment groups in both males and females generally clustered by removal or duration change treatments. Thus, prolonged (incubation period prolonged by 3 days) and extended treatments [prolonged by 3 days + a day with nestling(s)] formed a distinct cluster whereas all removal groups and the reduced incubation period group formed another cluster. The inclusion of the shortened incubation period group likely represents similarities in the hormone levels at mid-incubation and a limitation in hormone response due to internal timing.

## Conclusions

Overall, our data support previous descriptive work that characterized the profiles of CORT, progesterone, estradiol, testosterone, and prolactin across the reproductive cycle in birds. In addition, we find evidence of covariation between prolactin and CORT in females across breeding stages. We also find that hormone profiles tended to be more similar in early *vs*. late sampling stages in both males and females. Using experimental manipulations, we showed sex-specific hormone responses to nest loss, and to increased and decreased incubation period and extended hatching. Males increase sex steroid levels shortly after nest loss while females do not. We suggest that this responsiveness in males may help stimulate females to return to breeding condition more quickly. These data add to the growing body of data that suggest sex-specific differences in investment strategy following perturbations. This is the first in a set of papers from this group that will describe parental endocrine and genomic changes over parental care and in response to experimental manipulation.

## Data Availability Statement

The raw data supporting the conclusions of this article will be made available by the authors, without undue reservation.

## Ethics Statement

The animal study was reviewed and approved by UC Davis Institutional Animal Care and Use Committee.

## Author Contributions 

RC conceived the idea, and RC and MM provided funding. SA contributed to the experimental design (adding several sampling points) and conducted the data collection with CL, AB, VF, AMB, and JK. SA and JK extracted the hormones and validated and conducted the corticosterone, progesterone, estradiol, and testosterone RIAs in the Wingfield lab. RV assisted with hormone extractions and the aforementioned RIAs. JK and JW provided technical assistance and expertise. FA conducted the prolactin assay. SA analyzed the data and drafted the manuscript. All authors contributed to the article and approved the submitted version.

## Funding

This work was funded by NSF IOS 1455960 (to RC and MM).

## Conflict of Interest

The authors declare that the research was conducted in the absence of any commercial or financial relationships that could be construed as a potential conflict of interest.

## Publisher’s Note

All claims expressed in this article are solely those of the authors and do not necessarily represent those of their affiliated organizations, or those of the publisher, the editors and the reviewers. Any product that may be evaluated in this article, or claim that may be made by its manufacturer, is not guaranteed or endorsed by the publisher.
