# Supplementary material for: Uncovering the Sex-Specific Endocrine Responses to Reproduction and Parental Care
Source: Front Endocrinol (Lausanne). 2021 Nov 11;12:631384. doi: 10.3389/fendo.2021.631384 (PMC8632640; doi:10.3389/fendo.2021.631384)
Supplement: Supplementary file 1 [file Table_1.docx]

**Supporting information:**

Table 1: Sample sizes for each hormone and sex across characterization and manipulation time points.
